# The Newcomb-Benford Law in Its Relation to Some Common Distributions

**DOI:** 10.1371/journal.pone.0010541

**Published:** 2010-05-07

**Authors:** Anton K. Formann

**Affiliations:** Department of Psychological Basic Research, University of Vienna, Vienna, Austria; John Innes Centre, United Kingdom

## Abstract

An often reported, but nevertheless persistently striking observation, formalized as the Newcomb-Benford law (NBL), is that the frequencies with which the leading digits of numbers occur in a large variety of data are far away from being uniform. Most spectacular seems to be the fact that in many data the leading digit 1 occurs in nearly one third of all cases. Explanations for this uneven distribution of the leading digits were, among others, scale- and base-invariance. Little attention, however, found the interrelation between the distribution of the significant digits and the distribution of the observed variable. It is shown here by simulation that long right-tailed distributions of a random variable are compatible with the NBL, and that for distributions of the ratio of two random variables the fit generally improves. Distributions not putting most mass on small values of the random variable (e.g. symmetric distributions) fail to fit. Hence, the validity of the NBL needs the predominance of small values and, when thinking of real-world data, a majority of small entities. Analyses of data on stock prices, the areas and numbers of inhabitants of countries, and the starting page numbers of papers from a bibliography sustain this conclusion. In all, these findings may help to understand the mechanisms behind the NBL and the conditions needed for its validity. That this law is not only of scientific interest *per se*, but that, in addition, it has also substantial implications can be seen from those fields where it was suggested to be put into practice. These fields reach from the detection of irregularities in data (e.g. economic fraud) to optimizing the architecture of computers regarding number representation, storage, and round-off errors.

## Introduction

Newcomb [1] observed how much faster the first pages of tables of decadic logarithms wear out than the last ones, indicating that the first significant figure is oftener 1 than any other digit, and that the frequency diminishes up to 9. Without giving actual numerical data and a strict formal proof, he reached the conclusion that “The law of probability of the occurrence of numbers is such that all mantissae of their logarithms are equally probable”, so that “every part of a table of anti-logarithms is entered with equal frequency”. This resulted in a table giving the probabilities of occurrence in the case of the first two significant digits; see Table 1.

**Table 1:** Probabilities of occurrence for first four digits according to the Newcomb-Benford law.

	Digit									
Place	0	1	2	3	4	5	6	7	8	9
1.	–	3010	1761	1249	0969	0792	0669	0580	0512	0458
2.	1197	1139	1088	1043	1003	0967	0934	0904	0876	0850
3.	1018	1014	1010	1006	1002	0998	0994	0990	0986	0983
4.	1002	1001	1001	1001	1000	1000	0999	0999	0999	0998

Notes: Decimal points omitted. First and second digits' probabilities as given in Newcomb(1881), third and fourth digits' probabilities supplemented.

More than a half century later, Benford [2] rediscovered Newcomb's observation. Based on substantial empirical evidence from 20 different domains, such as the surface areas of 335 rivers, the sizes of 3259 U.S. populations, 104 physical constants, 1800 molecular weights, 5000 entries from a mathematical handbook, 308 numbers contained in an actual issue of Readers' Digest, the street addresses of the first 342 persons listed in American Men of Science, and 418 death rates, Benford stated a logarithmic law of frequencies of significant digits. This law gives
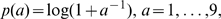
(1)for the probability 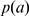 of the digit 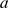 in the first place of observed numbers and
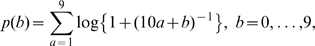
(2)for the probability 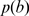 of the second-place digit 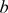. Most of the 20 domain-specific distributions of the first-place digits showed rather good agreement with the logarithmic law (1) – that later came to be known as Benford's or Newcomb-Benford law (NBL) –, but the averaged distribution fitted nearly perfectly.

These findings initiated “a varied literature, among the authors of which are mathematicians, statisticians, economists, engineers, physicists, and amateurs”, as Raimi [3] wrote in his comprehensive review on the first digit problem (p.521). After having described and discussed several approaches taken to ground the NBL, namely density and summability, scale-invariance, base-invariance, and mixture-distribution arguments, he concludes that – up to that time – Pinkham's [4] scale-invariance argument gave the first theoretical explanation of the NBL, however assuming a cumulative distribution function that cannot exist [5], pp.253–264, and assuring only a miserable numerical approximation. As an example, on p.533 Raimi [3] mentions the half Cauchy distribution with scale parameter *a* and density 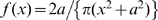, 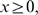 which satisfies all the relevant hypotheses stated by Pinkham. For this distribution, Pinkham's formula gives the lower and upper bounds .05 and 0.55, respectively, for the first-place digit to be 1. But some 15 years earlier, Furry and Hurwitz [6] already had derived much more precise bounds for the half Cauchy distribution.

Since Raimi's [3] review, the literature on the NBL has been expanded considerably. This can be seen from the bibliography compiled by Hürlimann [7] in 2006 with its 350 entries and from the up-to-date online bibliography implemented by Berger and Hill [8] that actually lists nearly 600 sources related to the NBL. Major theoretical advances have to be attributed to Hill who showed in a series of papers that base-invariance implies the NBL [9], [10], and that random samples coming from many random distributions may generate a compound distribution fulfilling the NBL [11]. Schatte [12] and Lolbert [13] studied the NBL in dependence on the numeral base, with the result that the approximation by the NBL becomes worse outside a limited range of bases. The relationship between the distribution of first digits with Zipf's law, with prime numbers and Riemann zeta zeroes, and with order statistics was investigated by Irmay [14], Luque and Lacasa [15], and Miller and Nigrini [16], respectively. Further, it was shown that exponential random variables [17] and other survival distributions [18] obey the NBL, that mixtures of uniform distributions fulfill a (generalized) version of the NBL [19], [20], and that data coming from different types of multiplicative processes also result in a first-digit distribution following the NBL [21]–[23] (but see also earlier results in [24], [25]) as do geometric sequences, for example powers of two [3], p.525. Bounds for the approximation error to the NBL were given by Dümbgen and Leuenberger [26] for the (half-)normal, the log-normal, the Gumbel, and the Weibull (including the exponential) distributions.

The NBL has been shown to fit rather closely many empirical data: in addition to most of those analyzed by Benford [2], among others, stock index returns [27], stock prices [28], eBay auctions [29], and consumer prices half a year after the introduction of the Euro in 2002 [30]. In contrast, the latter study found deviations from the NBL due to psychological pricing (consumer prices preferably ending in 0, 5, or 9) immediately after and a full year after the introduction of the Euro. So, the NBL may be useful as a benchmark for detecting irregularities in data. This has become of widespread use in economic fraud detection (e.g. tax evasion) [31], but the NBL was also proposed as a means to identify possible problems with survey data [32], self-reported ratings [33], and scientific results [34]. Because of its prominence, the NBL found even entrance in esteemed newspapers [35]. Another, merely future field for putting the NBL into practice is computer design. Theoretical considerations concerned the interrelation between number representation and storage requirements, as well as round-off errors arising in the computation of products [10]. Interestingly, empirical evidence was provided by Torres et al. [23] that file sizes in PCs behave according to the NBL of the first and second digit. In all, the goal could be to optimize the architecture of computers in order to fasten precise calculations and to save storage, both by taking the implications of the NBL into account.

On the other hand, many data obey the NBL rather badly or simply not, for example some mathematical functions such as square roots and the inverse 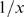. In his review, Raimi [3] gave two empirical examples for failure of the NBL: the 1974 Vancouver (Canada) telephone book, where no number began with the digit 1, and sizes of populations of all populated places with population at least 2500 from five US states according to the censuses from 1960 and 1970, where 19% only began with digit 1 but 20% with digit 2. To give but one recent empirical example, Beer's [36] finding should be mentioned that terminal digits of data in pathology reports do not follow the NBL. A simple explanation of the incompatibility of empirical data with the NBL cannot be found in any case, but these three cases have their obvious peculiarities: assignment of telephone numbers in an arbitrary manner, truncation of population size at 2500 inhabitants, and rounding data, comparable to psychological pricing of consumer goods.

It seems that nowadays the practical potentialities of the NBL have been recognized, and that meanwhile this empirically derived law can be considered theoretically well-analyzed. However, its relation to common distributions of random variables was investigated up to now only rudimentary [6], [17], [18], [26]. In addition, previous studies concentrated on the first digit, derived the deviation of the distribution under consideration from the NBL by calculating or approximating the respective integrals, and did not consider functions of random variables. In contrast, the present study investigates the leading ten digits and counts their frequencies from simulated data for different numbers of figures generated, whereby this is done not only for the random variables themselves, but also for ratios therefrom. This proceeding allows one to get an impression of the degree to which real data of finite sample size may approach the distribution predicted by the NBL while adopting one of Newcomb's arguments stated in the second paragraph of his two-pages 1881 note [1]: “As natural numbers occur in nature, they are to be considered as the ratios of quantities. Therefore, instead of selecting a number at random, we must select two numbers at random, and inquire what is the probability that the first significant digit of their ratio is the digit n. To solve the problem we may form an indefinite number of such ratios, taken independently;… (p.39).

This statement suggests the interpretation that Newcomb did not intend to consider numbers stemming from one and the same domain, for example from one of those investigated later by Benford, but that he had in mind to consider numbers drawn at random from the universe of all possible domains. If so, the measure 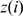 being available for an object 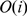 can be understood as the ratio 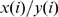 of the two numbers 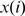 and 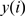, where 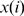 represents the object's size “per se” and 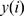 represents the scaling unit. In case that the objects stem from the same domain and were measured on the same scale, the scaling constant is no longer of interest and considering the measures 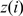 as given entities is appropriate, the more so as the Newcomb-Benford distribution has been shown to be scale-invariant. (This means that performing an admissible transformation of the ratio scale – that is, by multiplying all of the values by a positive constant – does neither reduce nor improve the degree to which the NBL fits the data.) Therefore, both relations are of interest: within one domain the relation between the NBL and the distribution of a random variable, and across domains the relation between the NBL and the ratio distribution of two random variables.

## Methods

**Figure 1:**
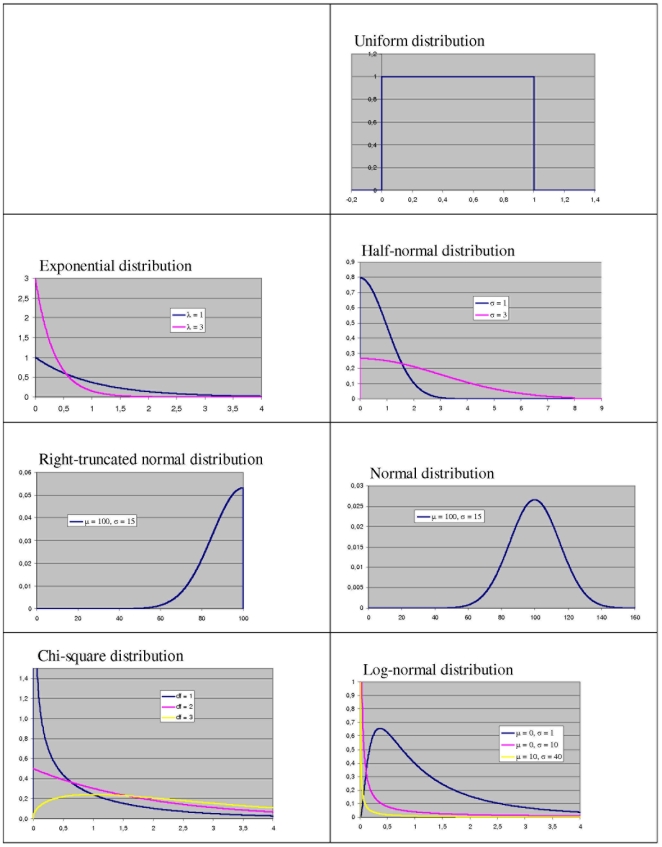
Seven common distributions of random variables. Uniform distribution, exponential distributions with 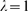 and 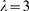, half-normal distributions with 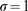 and 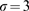, right-truncated normal distribution with 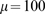 and 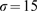, normal distribution with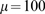 and 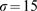, chi-square distributions with 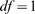, 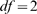, and 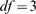, log-normal distributions with 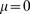 and 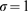, 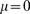 and 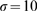, and 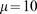 and 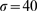.

**Figure 2:**
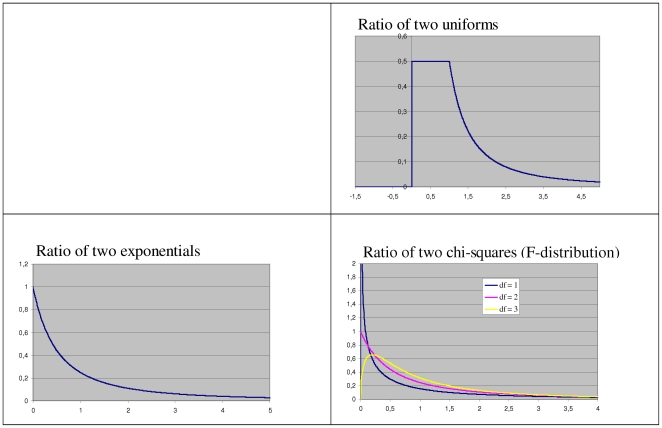
Three distributions of the ratio of two random variables. Ratio distribution of two uniforms *U*(0,1), ratio distribution of two exponentials with same 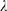, ratio distribution of two chi-squares (*F*-distribution) with 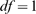, 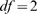, and 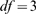.

Out of the manifold of common distributions seven were selected for the simulation study. Criteria for inclusion were, first, that each one of the distributions gives support for 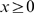 only, second, that some of the earlier investigated distributions should be included in order to allow comparisons, and, third, that across the selected distributions their shape should vary from right-skewed to left-skewed, including symmetric distributions. The seven types of distributions illustrated in Figure 1 and the resulting ratio-distributions (cf. Figure 2 for some of them) are the following ones.

- The **uniform distribution**, 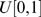, with density
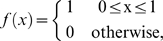
(3)merely as a test for the pseudo-random number generator, and in the case of ratios 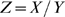, with 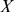 and 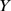 independent, because of the specific shape of the resulting density for 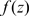,
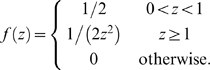
(3a)
- The **exponential distribution**, 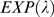, with density
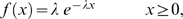
(4)as one of the survival distributions investigated earlier, so that for different sample sizes comparisons can be made with results obtained earlier [17], [18], [26]; if 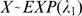 and 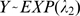, then, for 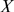 and 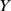 being independent, 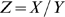
has density function
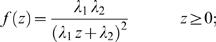
(4a)hence, for 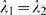 the density of 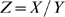 becomes independent of 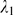 and 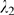 and has the simple form
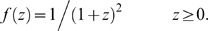
(4a′)
- The **half-normal distribution**, 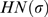, with density
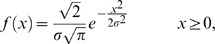
(5)thus also decreasing with increasing 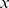, as a distribution not belonging to the classic survival distributions, in order to allow comparison with earlier results [6], [26]; the distribution of the ratio 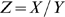, if 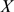 and 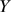 are independent and both follow the half-normal, results as a special case of two folded normals, the latter having a very complicated density; for details and some interrelations to other distributions, see Kim [37].
- The **right-truncated normal** with truncation at 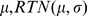 with density
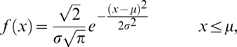
(6)to take into account a distribution whose density increases with increasing 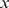; for sufficiently large 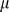 in relation to 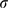, this distribution gives factual support for 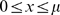 only; the distribution of the ratio of two independent right-truncated normals again results as a special case of two folded normals [37].
- The **normal distribution**, 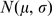
with density
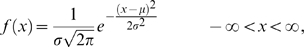
(7)which for sufficiently large 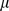 in relation to 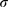 also gives factual support for 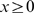 only, in order to include a very common member of the family of symmetric distributions; the distribution of the ratio 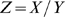, if 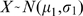 and 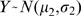 has a rather complicated form, which was derived by Hinkley [38] and which will not be given here.
- The **chi-square distribution** with 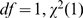, and density
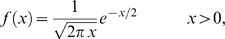
(8)the chi-square distribution with 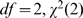, and density
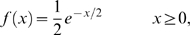
(8′)which, thus, equals that of the exponential with 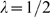 (cf.(4)), as well as some chi-square distributions with larger degrees of freedom; as is well-known, for 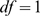 the chi-square distribution resembles a survival distribution, for increasing 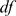 it approaches the normal distribution 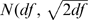); if 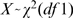 and 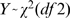, with 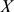 and 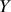 independent, then the ratio 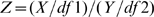 follows the *F*-distribution, 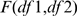, so that for 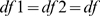, 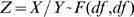; for 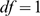, this ratio distribution has density
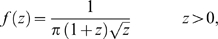
(8a)for 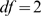 its density equals that of the ratio of two exponentials when 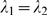, see (4a′); for 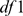 and/or 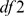 equal to 1 or 2, the *F*-distribution looks like a survival distribution, for increasing 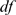 it tends to become symmetric around its mean.
- The **log-normal distribution**, 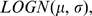 with density
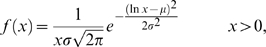
(9)because of its usage in cases where the random variable under consideration is thought of as the multiplicative product of many independent random variables (cf. the Introduction, where it was mentioned that multiplicative processes were shown to result in the Newcomb-Benford distribution, and [26]); as a function of 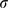, the log-normal distribution exhibits a behaviour similar to that of the chi-square and *F*-distributions, varying between long right-tailed to symmetric around its mean; if 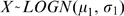 and 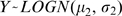, then for 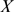 and 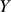 independent, the product 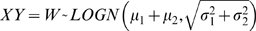, see Johnson, Kotz and Balakrishnan [39], p.216, so that the ratio 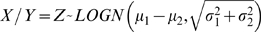. Note that the ratio distribution of two log-normals with 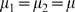 becomes independent of 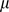.

According to each one of these distributions random numbers 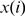 were generated for increasing sample size, 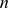, beginning with 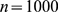 up to 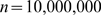. For the ratio distributions 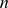 pairs of random numbers 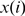 and 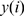 were generated, from which the 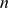 ratios 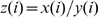 were calculated, that is, the ratio distributions were not involved directly. This can be seen to be an advantage of the simulation approach: in principle, the distribution of the ratio 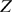 of two (independent) random variables 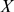 and 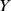 can be generated in that way for any two distributions of 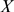 and 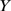, even without knowing the form of the distribution of 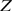. To save space, results will not be presented for all sample sizes under study, but mostly for 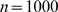 (realistic sample size for real data) and 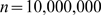 (to approximate the true distributions). In the next step, the frequencies of the first ten leading digits were counted. As for the sample sizes, results will be given in a reduced manner, namely for the first- and the second-place digits only. (No drastic irregularities became observable for third-place etc. digits. Moreover, it is known since Newcomb [1] that already the distribution of the third-place digit follows rather closely the uniform; see Table 1.) All of the calculations were performed in double precision by a FORTRAN program using the built-in function RANDOM which produces uniformly distributed pseudo-random variables between 0 and 1.

## Results

The numerical results for the **uniform distribution** and the ratio distribution of two uniforms are shown in Table 2. The uniform distribution produces a uniform distribution of first- and second place digits, as was to be expected. Hence, the clear conclusion is, that the uniform distribution and the NBL are incompatible. Nevertheless it is instructive to consider in more detail the discrepancies between the simulated relative frequencies of the digits and their theoretical values to get an impression of the precision which can be expected from the simulation study. Assuming a uniform distribution, the probabilities of occurrence for the first-place digits are 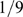 and for the second-place digits they are 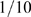. For the first-place digit, the deviation of the simulated relative frequencies from these values does not exceed .0231 for 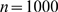 and .0013 for 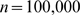, respectively. Similar maximal discrepancies (.0250 for 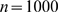 and .0018 for 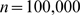) are obtained for the second-place digit. Nearly perfect agreement is found for the first two digits and 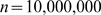, that is, under this sample size the true distribution is generated nearly perfectly. Across all sample sizes, for each digit 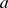 its simulated relative frequency 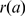 lies within the approximate (for the number of tests corrected overall) 99% confidence interval around the corresponding probability 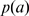, CI: 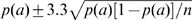. From this it can be concluded that the pseudo-random number generator works properly. Therefore, rather reliable results can be expected for all distributions under study even for 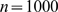 in terms of absolute differences between simulated and true distributions. But in terms of relative differences 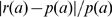, the agreement must be expected to be much weaker: the maximal relative differences turn out to be 20.29% for 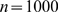 and 1.17% for 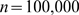 in the case of the first-place digit, and 25.00% for 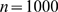 and 1.80% for 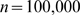 in the case of the second-place digit. One has to bear in mind these facts when evaluating the fit to the NBL in the presence of real data with moderate sample size, as well as when interpreting the results for the various distributions in the following.

**Table 2:** Uniform distribution and ratio of two uniforms.

		Digit									
Place		0	1	2	3	4	5	6	7	8	9
Uniform *U*[0,1]											
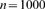	1.	–	0880	1080	1140	0950	1290	1210	1170	1290	0990
	2.	0930	0990	0900	0840	0910	0980	1250	1190	1100	0910
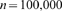	1.	–	1120	1117	1110	1102	1124	1106	1113	1100	1108
	2.	1001	1005	1001	1004	1004	1016	0995	1001	0982	0992
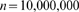	1.	–	1111	1111	1111	1111	1112	1111	1112	1111	1109
	2.	1001	1000	1000	1000	0999	1000	0998	1000	1001	1001
Ratio of two uniforms *U*[0,1]											
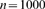	1.	–	3450	1140	1120	0640	0640	0900	0690	0640	0780
	2.	1240	1110	0990	1040	1060	1000	0900	0890	0870	0900
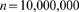	1.	–	3332	1481	1019	0834	0741	0688	0655	0634	0617
	2.	1293	1191	1106	1044	0988	0943	0903	0872	0842	0818

In contrast to the uniform distribution, the **ratio distribution of two uniforms** fits the NBL rather good. For 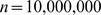 the maximal absolute difference between the simulated relative frequencies and the probabilities according to the NBL amounts to .0322, is found for the leading digit 1, and corresponds to a relative difference of 10.7%. For the same sample size even larger relative differences are found in some cases. For example, for the leading digit to be 9, the absolute difference is .0159 only, however resulting in the relative difference of nearly 35%. Especially for the large sample size most of the simulated relative frequencies fall outside any usual confidence interval around the digits' probabilities as given by the NBL. Thus, the NBL does not hold in a strict sense for the ratio distribution of two uniforms, that is, for unrealistically large sample sizes the H0: “The digits' distributions follow the NBL” would have to be rejected. But the NBL approximates the digits' distributions to such a degree that it may be acceptable as a H0 in the presence of real data sets with typical sample size.

The numerical results for the **exponential distribution** with parameter 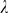 = 0.5, 1, 2 and the ratio distribution of two exponentials are given in Table 3. The exponential distribution produces first- and second-place digits' distributions coming close to the Newcomb-Benford distribution. As derived theoretically by Engel and Leuenberger [17] and also shown numerically by Leemis, Schmeiser and Evans [18], however for the leading digit only, the maximal absolute deviation is less than 0.03. This result was reproduced here, and it does not only apply to large samples, but also to the sample size of 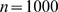. Further, it generalizes to the second-place digit. Note that for both the first- and second place the quality of fit depends on 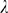 and varies across the digits.

**Table 3:** Exponential distribution and ratio of two exponentials.

		Digit									
Place		0	1	2	3	4	5	6	7	8	9
Exponential 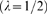											
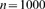	1.	–	2900	2010	1200	1030	0690	0800	0380	0480	0510
	2.	1160	1230	0890	0970	1040	1080	0970	0870	0990	0800
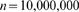	1.	–	2971	1945	1353	0987	0757	0612	0516	0451	0408
	2.	1171	1122	1082	1041	1008	0974	0943	0914	0885	0860
Exponential 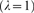											
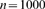	1.	–	3210	1720	1180	0990	0910	0540	0580	0320	0550
	2.	1230	1050	1160	1270	1100	0840	0850	0880	0770	0850
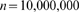	1.	–	3298	1744	1128	0860	0725	0642	0582	0532	0489
	2.	1210	1153	1100	1051	1006	0964	0929	0893	0862	0833
Exponential 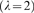											
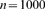	1.	–	2900	1900	1120	0870	0810	0900	0550	0520	0430
	2.	1120	1120	1000	1010	0890	1030	1110	0920	0870	0930
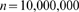	1.	–	2872	1585	1225	1020	0869	0744	0641	0558	0486
	2.	1212	1147	1089	1041	0999	0961	0928	0899	0873	0851
Ratio of two exponentials with parameters 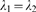											
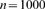	1.	–	3160	1560	1370	0970	0740	0730	0460	0470	0540
	2.	1180	1290	1080	1070	1020	0900	0980	0790	0770	0920
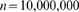	1.	–	3021	1752	1244	0965	0791	0670	0582	0516	0460
	2.	1198	1140	1088	1044	1004	0968	0933	0903	0873	0849

The **ratio distribution of two exponentials** with 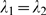 clearly outperforms these results. For 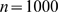, the maximal absolute deviation amounts to .0150 for the first-place digit to be 1 and to .0151 for the second-place digit also to be 1; for 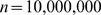, the maximal deviation is found to be .0011 (first-place digit 1). Most simulated relative frequencies look as if they were generated under the NBL, and they lie within the confidence intervals introduced above, except for 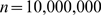. Comparable results were obtained for some ratio distributions of exponentials with 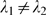, but details will be omitted.

The numerical results for the **half-normal distribution** with 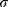 = 1, 2.5, 5 and the ratio distribution of two half-normals are shown in Table 4. The three half-normals under investigation do not fit the NBL as well as was to be expected following Dümbgen and Levenberger [26], but far better than given by Furry and Hurwitz [6]. According to our results, the maximal deviance across all cases studied is found to be .0790 for the first-place digit to be 1 if 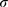 = 1 and 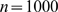, whereas Furry and Hurwitz reported .33. (Note that Furry and Hurwitz speak of the normal distribution, in fact they investigated the half-normal, as can be seen from their formula (a) on p.53. Note further that they reported .115 for the deviance of the exponential distribution – which now is known to be much smaller, see above –, but .0557 for the half Cauchy distribution that was not included in the present study because of its similarity with the normal distribution; cf. thereto p.300 in Johnson, Kotz and Balakrishnan [39]). The digits' distributions remain unaffected when multiplying 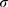 by integer powers of 10 so that, for example, the entries found in the first half of Table 4 also apply to the half-normal with 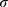 = 10, 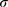 = 25, and 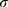 = 50, respectively.

**Table 4:** Half-normal distribution and ratio of two half-normals.

		Digit									
Place		0	1	2	3	4	5	6	7	8	9
Half-normal ( 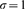 )											
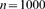	1.	–	3800	1190	0800	0680	0640	0750	0810	0850	0480
	2.	1270	1120	0930	1200	0940	0930	0970	0920	0960	0760
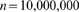	1.	–	3636	1279	0850	0803	0769	0732	0687	0644	0600
	2.	1263	1190	1124	1062	1007	0954	0907	0864	0830	0798
Half-normal ( 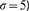											
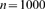	1.	–	2880	2390	1620	1120	0500	0480	0320	0300	0390
	2.	1100	1160	1080	0980	0900	1050	1090	0860	0810	0970
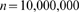	1.	–	2991	2299	1579	1002	0638	0450	0370	0341	0330
	2.	1135	1104	1075	1043	1012	0984	0956	0926	0894	0870
Half-normal ( 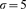 )											
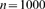	1.	–	1990	1320	1560	1330	1060	0880	0740	0630	0490
	2.	1220	1010	1100	1180	0980	0900	0950	0780	0950	0930
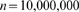	1.	–	2129	1572	1419	1243	1056	0873	0706	0559	0443
	2.	1196	1116	1059	1016	0984	0962	0940	0923	0908	0897
Ratio of two half-normals with 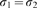											
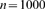	1.	–	3170	1590	1110	0880	0820	0660	0650	0530	0590
	2.	1370	1240	0980	0940	1070	0870	0960	0830	0990	0750
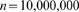	1.	–	3099	1685	1182	0938	0787	0683	0604	0539	0484
	2.	1211	1149	1094	1045	1001	0963	0928	0897	0868	0842
Ratio of two half-normals with 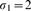 and 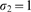											
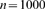	1.	–	3250	2000	1170	0840	0750	0590	0520	0490	0390
	2.	1100	1360	1140	0940	0960	0870	0990	0890	0810	0940
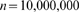	1.	–	3097	1845	1254	0936	0749	0632	0550	0491	0447
	2.	1190	1136	1089	1045	1005	0970	0935	0906	0876	0848
Ratio of two half-normals with 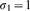 and 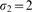											
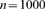	1.	–	2700	1700	1310	1120	1060	0660	0510	0430	0510
	2.	1260	1130	0950	1020	0920	0970	0890	1110	0920	0830
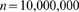	1.	–	2866	1725	1287	1023	0838	0699	0594	0515	0453
	2.	1195	1136	1083	1041	1001	0968	0933	0906	0880	0857

Surprisingly good fit to the NBL shows the **ratio distribution of two half-normals** with 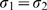 (independent of their actual values), 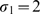, 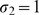, and 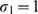, 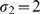. The fit is not as perfect as it is for the ratio of two exponentials, but it is better than that of the ratio of two uniforms. Especially good agreement is observed for the second-place digit under all three scenarios studied here, and even for the first-place digit the maximal deviance is found to be only .0089, .0087, and .0256, respectively (digit 1, 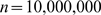). Overall, it seems to make little difference of whether the variances of the two random variables are equal or not, with the slight tendency to worsen the fit if the variance of the variable in the denominator, 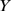, exceeds that of the numerator, 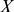, in the ratio 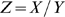.

The numerical results for the **right-truncated normal distribution** and the **ratio distribution of two right-truncated normals** are given in Table 5. Its entries speak for themselves so that a short comment will suffice. As compared with survival distributions, the right-truncated normal shows inverse behaviour in that it puts most mass on large values of the random variable. That is why the right-truncated normal was selected for inclusion in the present study. It turns out that it may serve as a prototypical example of distributions of random variables not leading to first- and second-place digits' distributions obeying the NBL. Presented are the figures only for 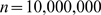, two distributions, with 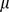 = 1.1, 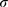 = 0.25 and 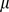 = 100, 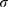 = 15, and their ratio distributions. The discrepancies between the simulated digits' distributions and the Newcomb-Benford distribution are such that even for small sample sizes conventional goodness-of-fit tests, for example Pearson's chi-square and the likelihood-ratio test, have a good chance to become significant. Considering the ratio distribution of two right-truncated normals does not improve matters. (Note that nonconformance to the NBL was reported for the Gumbel distribution whose density also increases with increasing value of the random variable [26].)

**Table 5:** Right-truncated normal distribution and ratio of two right-truncated normals.

		Digit									
Place		0	1	2	3	4	5	6	7	8	9
Right-truncated normal ( 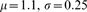 )											
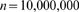	1.	–	3075	0008	0033	0110	0295	0657	1232	1950	2641
	2.	3637	0589	0619	0649	0677	0705	0736	0767	0796	0826
Right-truncated normal ( 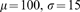 )											
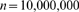	1.	–	0000	0000	0000	0005	0061	0381	1402	3246	4905
	2.	0764	0815	0868	0919	0972	1028	1080	1132	1186	1236
Ratio of two right-truncated normals, both with 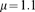 and 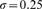											
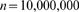	1.	–	4891	0104	0033	0081	0214	0472	0883	1399	1922
	2.	2204	1597	1191	0939	0789	0704	0661	0641	0635	0640
Ratio of two right-truncated normals, both with 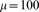 and 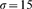											
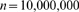	1.	–	4999	0002	0000	0001	0020	0132	0550	1495	2801
	2.	2957	1710	1022	0708	0591	0561	0568	0594	0627	0662

Similar results were obtained for the **normal distribution** and the **ratio distribution of two normals**; see Table 6. The normal distribution, putting most mass around the mean of the random variable, was selected for inclusion in the present study as a further possible candidate of nonconformity with the NBL. Neither the normal distribution nor the ratio distribution of two normals disappointed this expectation. As for the right-truncated normal, figures are presented for 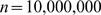 and two sets of parameters only.

**Table 6:** Normal distribution and ratio of two normals.

		Digit									
Place		0	1	2	3	4	5	6	7	8	9
Normal distribution ( 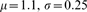 )											
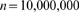	1.	–	6536	0005	0016	0055	0148	0329	0617	0975	1320
	2.	1820	1831	1630	1299	0954	0682	0515	0438	0414	0417
Normal distribution ( 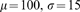 )											
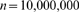	1.	–	4999	0000	0000	0003	0030	0191	0702	1623	2453
	2.	2834	2031	1134	0649	0516	0517	0541	0566	0594	0618
Ratio of two normals, both with 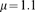 and 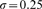											
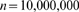	1.	–	4758	0230	0072	0179	0419	0749	1064	1254	1275
	2.	1663	1470	1266	1083	0936	0830	0750	0699	0664	0641
Ratio of two normals, both with 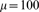 and 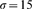											
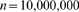	1.	–	4988	0011	0001	0011	0096	0398	0993	1615	1887
	2.	2143	1741	1313	0986	0779	0661	0607	0586	0590	0594

The numerical results for the **chi-square distribution** and the ratio distribution of two chi-squares are shown in Table 7. Regarding the chi-square distribution, a clear tendency becomes obvious. Very good fit to the NBL is found for the chi-square with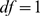 (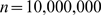, maximal deviance .0065 for first-place digit 2), increasing the 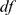 (shown for 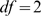 and 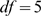) worsens the fit considerably. This does not come as a surprise when taking the shape of the chi-square distribution into account: the chi-square with 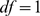 behaves like a survival distribution, for increasing 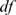 it approaches a normal distribution.

**Table 7:** Chi-square distribution and ratio of two chi-squares (*F*-distribution).

		Digit									
Place		0	1	2	3	4	5	6	7	8	9
Chi-square ( 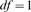 )											
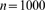	1.	–	3050	1840	1250	0850	0760	0820	0600	0500	0330
	2.	1330	1050	1070	1000	0950	0960	0960	0920	0820	0940
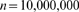	1.	–	3071	1826	1257	0949	0759	0639	0556	0495	0448
	2.	1192	1138	1087	1045	1006	0968	0936	0905	0874	0849
Chi-square ( 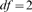 )											
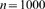	1.	–	2810	2090	1220	1020	0960	0620	0420	0520	0340
	2.	1200	1320	1270	0990	0860	0970	0960	0940	0850	0640
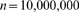	1.	–	2961	1959	1365	0993	0757	0607	0511	0445	0402
	2.	1168	1121	1079	1041	1007	0974	0944	0915	0888	0862
Chi-square ( 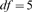 )											
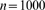	1.	–	1820	1580	1530	1440	1020	1080	0520	0480	0530
	2.	1220	1210	1040	0880	0960	0940	1140	0880	0840	0890
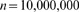	1.	-	1820	1494	1532	1383	1159	0927	0720	0547	0418
	2.	1183	1107	1052	1010	0983	0961	0945	0930	0920	0909
Ratio of two chi-squares, both with 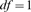											
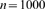	1.	–	3160	1510	1470	0770	0780	0770	0610	0450	0480
	2.	1170	1340	1100	1090	0960	0840	0950	0860	0880	0810
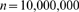	1.	–	3013	1760	1249	0969	0791	0667	0581	0512	0458
	2.	1196	1140	1089	1042	1003	0967	0935	0903	0874	0850
Ratio of two chi-squares, both with 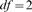 (see ratio of two exponentials, Table 3)											
Ratio of two chi-squares, both with 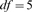											
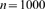	1.	–	3100	1610	1110	1090	0740	0670	0590	0520	0570
	2.	1030	1280	0990	1070	1050	1000	1070	0880	0870	0760
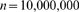	1.	–	3180	1619	1119	0907	0784	0698	0624	0562	0507
	2.	1221	1157	1100	1046	1004	0960	0924	0892	0860	0835

The **ratio distribution of two chi-squares (** ***F*** **-distribution)** with 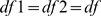 fits better than does the chi-square. Moreover, the ratio distribution of two chi-squares proves more robust against increasing the 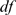. For 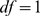, the simulated first- and second-place digits' distributions are nearly indistinguishable from the Newcomb-Benford distribution, and up to 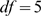 the deviance increases rather slowly. Note that the *F*-distribution with 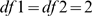 is formally identical to the ratio distribution of two exponentials with 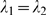. Therefore, figures for 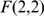 were omitted; see Table 3.

The numerical results for the **log-normal distribution** are given in Table 8. For this two-parameter distribution, the fit to the NBL heavily depends on 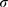 and slightly depends on 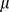. The larger 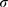 and/or 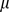, the better is the fit. For 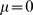, 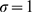 and 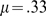, 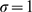 the misfit is massive, so that considering the effect of sample size becomes obsolete; hence figures are given for 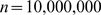 only. The best fit amongst the cases reported here is obtained with 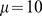, 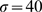: the simulated first- and second-place digits' distributions come very close to the Newcomb-Benford distribution when 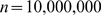; the maximal deviance amounts to .0064 and refers to the first-place digit 1. As the ratio distribution of two log-normals also follows the log-normal, no separate presentation of results is needed.

**Table 8:** Log-normal distribution.

		Digit									
Place		0	1	2	3	4	5	6	7	8	9
Log-normal ( 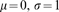 )											
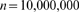	1.	–	6931	3069	0000	0000	0000	0000	0000	0000	0000
	2.	1442	1335	1246	1167	1098	1037	0983	0639	0541	0512
Log-normal ( 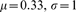 )											
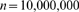	1.	–	3599	4054	2346	0000	0000	0000	0000	0000	0000
	2.	0816	0782	0752	0756	1387	1320	1256	1186	0893	0853
Log-normal ( 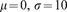 )											
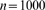	1.	–	3520	1680	1290	0980	0700	0590	0460	0440	0340
	2.	1200	1040	1160	1100	1080	1040	0840	0870	0880	0790
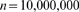	1.	–	3466	1719	1151	0893	0729	0616	0534	0472	0420
	2.	1248	1181	1083	1034	0994	0954	0922	0889	0861	0834
Log-normal ( 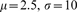 )											
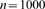	1.	–	3270	1690	1380	0940	0780	0600	0560	0390	0390
	2.	1130	1150	0970	0990	0880	1040	1020	1000	0860	0960
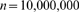	1.	–	3268	1915	1152	0892	0729	0617	0533	0471	0422
	2.	1151	1093	1112	1077	1034	0995	0953	0890	0861	0835
Log-normal ( 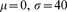 )											
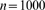	1.	–	2940	1710	1360	1010	0750	0780	0490	0450	0510
	2.	1250	1240	1230	1100	0930	0900	0930	0980	0740	0700
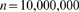	1.	–	3118	1764	1222	0951	0775	0655	0566	0501	0448
	2.	1206	1148	1096	1046	0997	0963	0929	0899	0871	0844
Log-normal ( 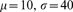 )											
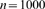	1.	–	2940	1900	1150	0890	0740	0690	0680	0510	0500
	2.	1380	1270	1020	1050	1100	0970	0730	0840	0890	0750
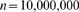	1.	–	2946	1801	1294	1003	0785	0654	0567	0501	0449
	2.	1190	1133	1089	1047	1004	0967	0935	0907	0877	0852

The results of the simulation study may be summarized in two statements. First, all types of distributions which turned out to be compatible with the NBL exhibit a common feature. They are long right-tailed and, thus, put most mass on small values of the random variable. To these distributions belong the exponential, the chi-square with very small degrees of freedom (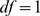 and 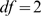), the log-normal with large variance, and, with some limitations, the half-normal. Incompatibility with the NBL proved the uniform, the normal, and the right-truncated normal distributions. Second, the fit to the NBL generally improves when considering distributions of ratios of random variables. Among the seven types of ratio distributions studied here, five emerged as being consistent with the NBL. The ratio distribution of two exponentials, the ratio distribution of two chi-squares (*F*-distribution) with small degrees of freedom, and the ratio distribution of two log-normals with large variance fitted the first- and second-place digits' distributions as given by the NBL nearly perfectly, the ratio distributions of two uniforms and of two half-normals fitted it sufficiently well, whereas only the ratio distributions of two normals and of two right-truncated normals completely failed to fit.

Together with findings reported earlier [6], [17], [18], [26] regarding the conformance to the NBL for some survival distributions (exponential, Muth, Gompertz, Weibull, gamma, log-logistic, and exponential power distributions) our results indicate that the validity of the NBL requires that the frequency of ‘natural’ numbers in the sense of Newcomb [1] decreases with increasing magnitude. Roughly speaking, this means that small numbers have to be predominant. That is, when thinking of real-world data, conformity to the NBL necessitates a majority of small objects. As the NBL has often been shown to be valid, conversely it can be deduced that, at least within numerous domains of our world, small objects must occur much more frequently than do large ones. Some examples given in the following will sustain this conclusion.

Analyzed were the distributions of the following five variables plus their first- and second-place digits' distributions.

- The closing prices in Euro as of June 30, 2009 of stocks contained in the AEX (Netherlands), ATX (Austria), CAC40 (France), DAX (Germany), DJI (USA), DJStoxx50 (Europe) and SMI (Swiss), in total 179 values whereby stocks entered only once when they appeared twice, namely in one of the local European stock indices and in the overall European index DJStoxx50;
- the closing prices of these stocks, however in local currencies (Euro, US-$, Swiss Franc);
- the areas of 198 countries;
- the numbers of inhabitants of these 198 countries; and
- the starting page numbers of 225 papers referenced in the bibliography on the NBL compiled by Hürlimann [7].

**Figure 3:**
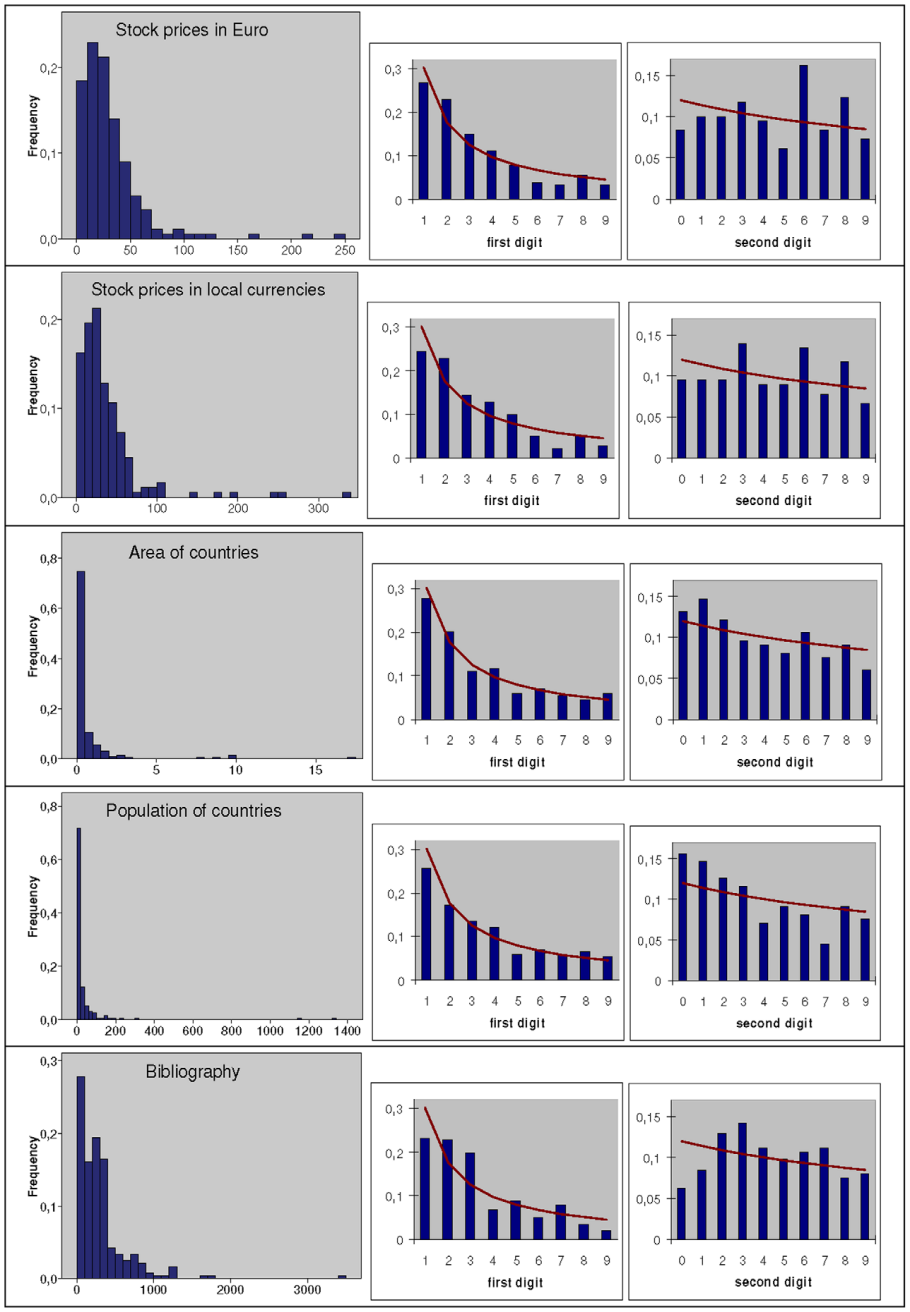
Five empirical examples. Stock prices in Euro, stock prices in local currencies, area of countries (in units of 100,000 sq.km.), population of countries (in units of millions), starting page of papers referenced in a bibliography on the Newcomb-Benford law. For each data set, the distribution of the observed variable is shown on the left, the resulting first- and second-place digits' distributions are shown on the right (bars) together with the respective distributions according to the Newcomb-Benford law (solid lines).

Overall, results are as expected. First, all five variables possess a marked majority of small and a clear minority of large realizations. Four of the five variables exhibit a distribution coming more (areas and inhabitants of countries) or less (stock prices: very low values are underrepresented) close to survival distributions. The distribution of one variable (the bibliography data) follows rather a step function than a continuously decreasing density function: the highest frequency is found for starting pages 1 to 99 as it was to be expected; the starting pages 100 to 199, 200 to 299, and 300 to 399 occur with markedly lower, but approximately constant frequency; then the frequency decreases sharply to a level remaining approximately constant for the following five 100-pages sections (Figure 3; frequency distributions on the left).

Second, in all five cases the first-place digit 1 is slightly underrepresented. Nevertheless, based on the Pearson chi-squared goodness-of-fit test (5% significance level), all of the first- and second-place digits' distributions are compatible with the NBL, with one exception: the first-place digit's distribution of the bibliography data clearly fails to fit the NBL; see Table 9. The best fit is found for the areas of countries and their numbers of inhabitants, weaker fit is found for both variants of stock prices (prices in Euro vs. prices in local currencies). Note that the second-place digit's distribution of the stock prices in Euro is a borderline case pointing at the importance not to look at the first-place digit only when testing for the fit of the NBL. The first- and second-place digits' distributions are shown in Figure 3 on the right, whereby observed values are represented by bars, values expected according to the NBL by a line.

**Table 9:** Results of the Pearson chi-square tests for the five empirical examples.

	First digit 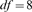		Second digit 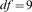	
	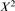	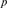	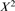	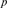
Stock prices in Euro	9.45	.306	16.94	.0497
Stock prices in local currencies	14.04	.081	10.20	.335
Area of countries	4.17	.841	5.39	.799
Inhabitants of countries	4.83	.776	11.70	.231
Bibliography	27.46	.001	14.06	.120

Third, and most importantly, the examples demonstrate the link between the distribution of a random variable on the one hand and the first-and second place digits' distributions on the other hand. The closer the shape of the distribution of a random variable comes to that of a survival distribution or a distribution behaving like a survival distribution, the better follows the first- and second-place digits' distributions the NBL. Regarding our five examples, the same ordering according to both properties is observable: the areas of countries and their numbers of inhabitants perform best, both versions of stock prices perform to some extent, but the bibliography data do simply not.

## Discussion

In the first part of this study seven types of common distributions were investigated regarding their conformance to the NBL. The results of the simulations showed first that all types of distributions behaving like survival distributions, that is, putting most mass on small values of the random variable and being long right-tailed, were compatible with the NBL. Second, distributions of the ratio of two random variables fitted better than did the distributions of a single random variable. For symmetric distributions (illustrated by example of the normal distribution), distributions tending to symmetry as a function of their parameters (illustrated by example of the chi-square and the log-normal distributions), and distributions whose density increases with increasing value of the random variable (illustrated by example of the right-truncated normal distribution), the misfit to the NBL was found to be substantial up to massive.

These observations together with the fact that the NBL – at least approximately – applies to many empirical data led to the suspicion that the size of ‘natural’ objects must follow a distribution behaving like a survival distribution in order to be able to obey the NBL. This suspicion could be substantiated by analyzing five sets of data. It turned out that the closer the distribution of a variable comes to that of a survival distribution the better is the fit to the NBL. Thereby, the fit to the NBL was tested formally by chi-square goodness-of-fit tests of the first- and second-place digits' distributions, whereas the fit of the observed variable's distribution to a survival distribution of unspecified form was informally assessed by visual inspection.

The overall conclusion resulting from the present study reads very simply. The frequently found good fit of the NBL to empirical data can be explained by the fact that in many cases the frequency with which objects occur in ‘nature’ is an inverse function of their size. Very small objects occur much more frequently than do small ones which in turn occur more frequently than do large ones and so on. Thus, the variable's distribution looks like a survival distribution whose leading digits' distributions follow the NBL, at least approximately.

It is somewhat surprising that in the literature on the NBL the connection between the distribution of a random variable and the leading digits' distributions was investigated up to now only for a handful of mainly survival distributions. Studies referring to empirical data concentrated solely on the leading digits' distributions, nearly always on the most significant digit only, neither discussing the relationship between the leading digits' distributions and the variable's distribution nor presenting the latter one. As a consequence, reanalyzing empirical data collected earlier was not possible and new data had to be found. Presumably the present study is therefore the first one focusing on the connection between the variables' distribution and the leading digits' distributions, in both theoretical and empirical settings. It remains to hope that future investigations on and applications of the NBL will pursue the approach taken here.
